# Malaria indicator survey 2009, South Sudan: baseline results at household level

**DOI:** 10.1186/1475-2875-13-45

**Published:** 2014-02-03

**Authors:** Margaret B Eyobo, Adwok C Awur, Gregory Wani, Ahmed I Julla, Constantino D Remijo, Bakhit Sebit, Robert Azairwe, Othwonh Thabo, Edward Bepo, Richard L Lako, Lul Riek, Emmanuel Chanda

**Affiliations:** 1 Management Sciences for Health, Juba, Republic of SouthSudan; 2 Ministry of Health, Juba, Republic of South Sudan; 3 National Bureau of Statistics, Juba, Republic of South Sudan; 4 Population Services International, Juba, Republic of South Sudan

## Abstract

**Background:**

South Sudan has borne the brunt of years of chronic warfare and probably has the highest malaria burden in sub-Saharan Africa. Malaria is the leading cause of morbidity and mortality in the country. This nationally representative survey aimed to provide data on malaria indicators at household level across the country.

**Methods:**

In 2009, data were collected using a two-stage random cluster sample of 2,797 households in 150 census enumeration areas during a Malaria Indicator Survey (MIS) in South Sudan. The survey determined parasite and anaemia prevalence in vulnerable population groups and evaluated coverage, use and access to malaria control services. Standardized Roll Back Malaria Monitoring and Evaluation Reference Group (RBM-MERG) MIS household and women’s questionnaires were adapted to the local situation and used for collection of data that were analysed and summarized using descriptive statistics.

**Results:**

The results of this survey showed that 59.3% (95% CI: 57.5-61.1) of households owned at least one mosquito net. The proportion of the population with access to an ITN in their household was 49.7% (95% CI: 48.2-51.2). The utilization of insecticide-treated nets was low; 25.3% (95% CI: 23.9-26.7) for children under five (U5) and 35.9% (95% CI: 31.9-40.2) of pregnant women (OR: 1.66 (1.36-2.01); P =0.175). Prevalence of infection was 24.5% (95% CI: 23.0-26.1) in children U5 and 9.9% (95% CI: 7.4-13.1) in pregnant women. About two thirds (64%) of children U5 and 46% of pregnant women were anaemic. Only 2% of households were covered by indoor residual spraying (IRS) the previous year. Data shows that 58% reported that malaria is transmitted by mosquitoes, 34% mentioned that the use of mosquito nets could prevent malaria, 41% knew the correct treatment for malaria, and 52% of the children received treatment at a health facility.

**Conclusion:**

The observed high malaria prevalence could be due to low levels of coverage and utilization of interventions coupled with low knowledge levels. Therefore, access and utilization of malaria control tools should be increased through scaling up coverage and improving behaviour change communication.

## Background

South Sudan has borne the brunt of years of chronic warfare and probably has the highest malaria burden in sub-Saharan Africa [1]. Malaria is the leading cause of morbidity and mortality in the country, accounting for 20 to 40% morbidity with over 20% of deaths reported at health facilities and 30% of all hospital admissions [2]. The disease is endemic countrywide putting the entire population at risk of infection and exacting a greater toll in children under five and pregnant women. Malaria endemicity varies from hypo-endemicity, through meso-endemicity, hyper-endemicity to holo-endemicity. Malaria transmission season peaks towards the end of the rainy season in September to November and is longer in the southern (seven to eight months) than in the northern (five to six months) regions [2].

Effective malaria control in post-conflict settings is hampered by a multiplicity of challenges and requires up to date and quality data to facilitate for rational deployment of interventions [1]. Following the signing of the Comprehensive Peace Agreement (CPA) in 2005, the South Sudan household and health survey was conducted in 2006 [3]. Recognizing the low coverage and use of malaria interventions in the country, the Ministry of Health (MoH) developed a National Malaria Control Strategic Plan for the period 2007–2012 [4]. Interventions included: early diagnosis with rapid diagnosis test (RDTs), treatment with artemisinin-based combination therapy (ACT), indoor residual spraying (IRS), long-lasting insecticidal nets (LLINs) and intermittent preventive treatment (IPTp) [4]. This was accompanied by information, education and behaviour change communication programmes.

Following an unprecedented increase in malaria control funding and the concomitant scale-up in deployment of interventions, several endemic countries have measured the coverage and impact of control tools [5]. This has been achieved largely through the nationally representative Malaria Indicator Surveys (MISs) using guidelines developed by the Roll Back Malaria (RBM) Monitoring and Evaluation Reference Group (MERG) [6]. However, no comprehensive malaria-specific survey was conducted in South Sudan to assess progress towards achieving national and global goals and targets. In 2009, a MIS was conducted to evaluate the coverage and impact of malaria control tools. The survey specifically aimed at assessing the coverage and use of key malaria control interventions, parasitaemia and anaemia in vulnerable populations, and the progress towards nationally and globally set goals.

## Methods

### Study population and approach

**Figure 1:**
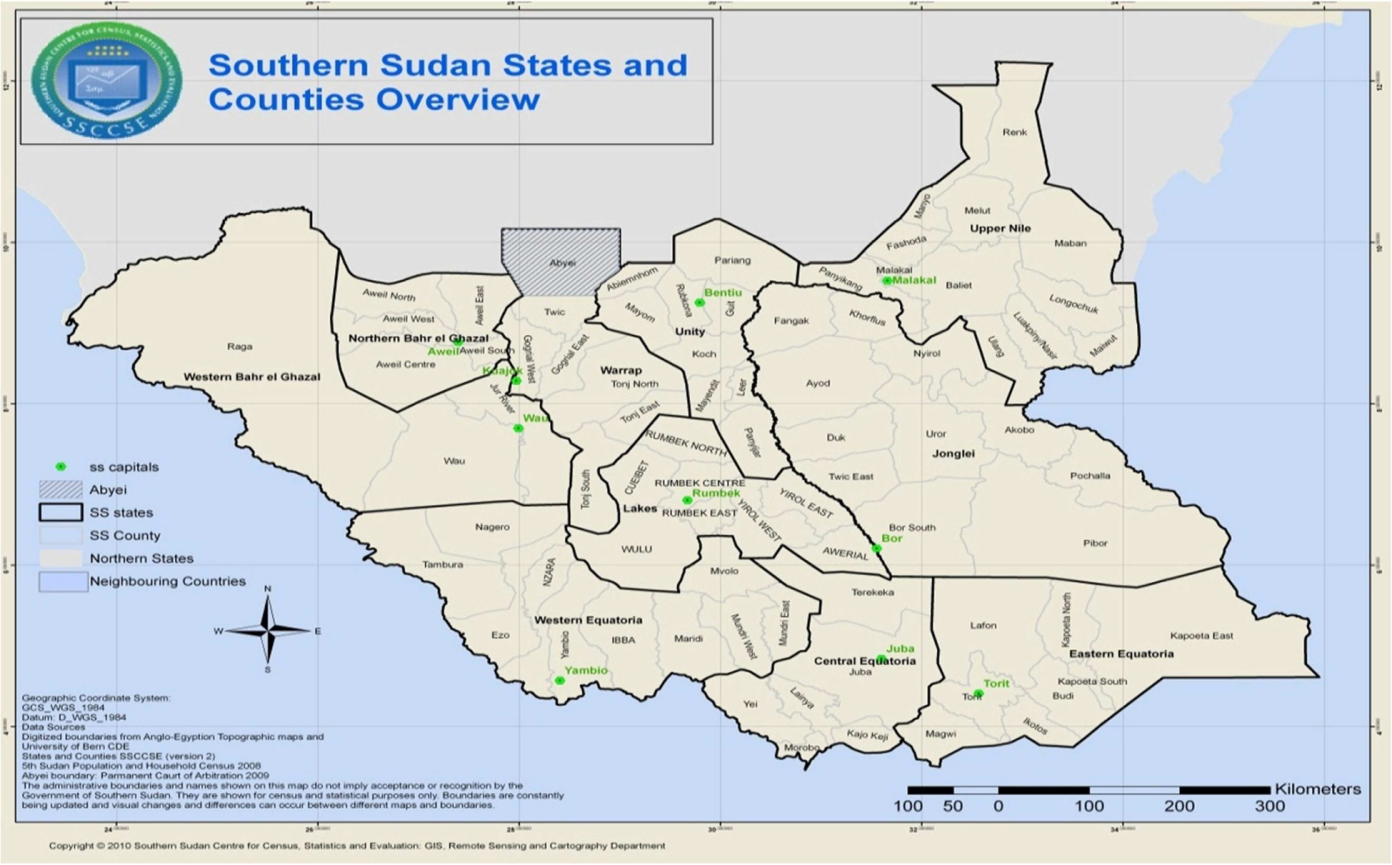
Map of South Sudan showing state boundaries (Source: SSCCSE, 2010).

South Sudan covers 650,000 sq km of land between 8° and 18° degrees south latitude and between 20° and 35°degrees east longitude (Figure 1) with a population of 8.3 million and almost 900,000 refugees, returnees and internally displaced persons [7]. While the population density is 13 p/sq km, only 17% of the total population resides in urban areas. South Sudan comprises ten states in three regions: 1) Greater Equatoria: Eastern Equatoria, Western Equatoria and Central Equatoria, 2) Greater Bahr el Ghazal: Western Bahr el Ghazal, Northern Bahr el Ghazal, Warrap and Lakes, and, 3) Greater Upper Nile: Unity, Upper Nile and Jonglei. The MIS was conducted between November and December 2009 in accordance with the RBM MERG protocol [6] adapted to local settings.

### Malaria control interventions

World Health Organization (WHO) recommended case management and vector control tools have been implemented expansively in South Sudan [4]. The WHO-led integrated vector management (IVM) has been adopted as the main approach for vector control. The NMCP developed a draft strategic plan for IVM for the period 2007–2012 [8]. The approach is to consolidate the use of LLINs while introducing additional interventions, i.e. IRS and larval source management (LSM), where applicable. Presently, the distribution of LLINs remains the only key operational vector control intervention with limited use of IRS and larviciding by Mentor Initiative, an NGO in Malakal County [9]. To date over 9.0 million LLINs have been distributed through mass distribution campaigns and health facility based routine distribution. The NMCP is putting in place implementation arrangements for operational deployment of targeted IRS and larviciding.

### Sample design

The survey was designed to provide nationally representative estimates of key malaria indicators and utilized a two-stage cluster sample design. The sample was stratified into three survey regions: Greater Equatoria, Greater Bahr el Ghazal and Greater Upper Nile. First, a total of 150 census enumeration areas (EAs) as primary sampling units, stratified by three domains and degree of urbanization, were selected with probability proportional to size and a complete listing of all households in each cluster was carried out. 50 clusters (EAs) were allocated to each of the three domains and stratification by urban/rural was done within the domains. The sample was proportionally allocated hence self weighting in the three strata. Second, 20 households per EA were selected for interviewing using equal probability systematic random sampling making a total sample of 3,000 households. To minimize potential bias, up to three visits were made to ascertain compliance in case of absence of all eligible respondents or subjects.

### Survey questionnaires

Standard MIS questionnaires based on the RBM MERG guidelines with modification to reflect relevant issues of malaria in South Sudan were used [6]. The household questionnaire was used to list all the usual members and visitors in the selected households and to identify eligible women for the individual interview and children aged 0–59 months for anaemia and malaria testing. It also collected basic information on the characteristics of each person listed, including age, sex, household's residence and assets, and ownership, type and use of mosquito nets. The woman’s questionnaire was used to collect information from all women aged 15–49 years on background characteristics, full reproductive history, prenatal care and preventive malaria treatment for most recent birth, prevalence and treatment of fever among children under five years, including knowledge about malaria causes, prevention and treatment.

### Survey organization, training and data collection

The survey designing, planning and implementation was a collaborative effort by multiple individuals from local and international malaria stakeholders. Prior to data collection, two levels of training, combining both course work and practicals, were conducted, a five-day central training of trainers (ToT) for the principal trainers, and ten-day state level cascade training for interviewers. Each interviewer had been given a detailed MIS manual, which was designed in accordance with WHO recommendations [6,10]. A total of 182 field staff participated in the survey. Surveyors were organized in 26 teams, each team consisted of a supervisor, three interviewers and three laboratory technicians and a driver. In addition, ten field operations managers, one per state, were recruited. All teams in a single state were coordinated through their supervisors and the state field operations manager, who collaborated with the central team in Juba.

### Malaria and anaemia diagnosis

The biomarkers in the survey included rapid diagnostic test (RDTs) and blood slides for microscopic examination for malaria and haemoglobin level testing for anaemia. Blood samples were collected from a finger-prick using a single-use, spring-loaded, sterile lancet. All the three tests were performed simultaneously from a single finger prick. Children aged 0–59 months and pregnant women were tested for anaemia because of the strong correlation with malaria infection. Haemoglobin concentration analysis was carried out on site using a battery-operated portable spectrophotometer (Hemo-Cue 201, Anglom, Sweden).

*Plasmodium falciparum* malaria testing was done using the Paracheck Pf™ RDT, which has shown good sensitivity and specificity in operational settings [11]. Test results for both RDT and anaemia were provided to the child’s parent/guardian verbally and were recorded on the household questionnaire. Two blood slides, thick and thin films, were taken for each participant by a laboratory technician as per standard WHO-approved protocol [12]. The blood slides were air-dried, fixed (thin films), stained with Giemsa and transported to Juba Teaching Hospital reference laboratory for reading. Based on standard laboratory malaria microscopy procedures, the microscopists determined the presence, density (thick blood film) and species of the malaria parasites (thin blood film). If no parasites were found after examination of 200 high power fields, the thick blood smear was considered negative and the corresponding thin blood film was not read. For external quality control, all positive blood slides plus 10% of the negative slides were sent for cross-checking at a WHO prequalified laboratory in the Republic of Oman. The parasite prevalence rates were harnessed for the production of Map of Epidemiological Stratification of *P. falciparum* malaria among U5s using geo-statistical modeling.

### Treatment

Children who tested positive for malaria using the RDT were offered a full course of treatment according to the standard protocol for treating malaria in South Sudan, i e, artesunate-amodiaquine-combination therapy (ACT) [13]. For children diagnosed with moderate-severe anaemia (ie, haemoglobin <8 g/dl), results were shared with the parent/guardian and the children were given artesunate-amodiaquine, albendazole (if >24 months of age as per national protocol for integrated maternal and child illnesses [13]) and supplemental iron. All severe cases with a positive RDT result and children with a haemoglobin level of under 8 g/dl were given a referral card and taken to a health facility for follow-up evaluation and treatment.

### Data management and analysis

Further to the successful collection of data during fieldwork, data processing staff were recruited and trained; these consisted of a supervisor from Southern Sudan Centre for Census Statistics and Evaluation (SSCCSE) and data entry operators. Data were entered twice using the CS Pro computer package and cleaned by checking missing cases and inconsistent entries. Descriptive statistics (ie, frequencies, percentages) were used to describe the characteristics of the sample and calculate coverage, use and access estimates. Data analysis was carried out in SPSS 16.0 (SPSS Inc, Chicago, IL, USA).

### Quality control

To ensure high quality data collection, the teams were visited daily by central and state supervisors and monitored by principal investigators during the survey period. The teams randomly inspected completed households to confirm correctness of records obtained from the survey and completion of supervisory checklist and observing a team’s overall performance as well as providing feedback and sharing the experiences of other teams. Additionally, the quality of data entering and analysis was performed by highly qualified statisticians.

### Ethical clearance

The survey protocol received ethical clearance from the South Sudan Ministry of Health Ethics Committee. Written or verbal, informed consent was obtained from the heads of households and each eligible individual before conducting the household questionnaires. Additional informed consent from a child’s parent or guardian and the pregnant women for blood films and anti-malarial treatment with ACT was provided by a nurse or physician when participants had a positive rapid test result.

## Results

### Attributes of sampled population

The MIS was conducted using a nationally representative sample of 3,000 households in150 enumeration areas. A total of 2,797 households were successfully interviewed. The data indicate that 17,000 people were enumerated in the survey with women constituting 52.0% (95% CI: 51.3-52.8) men 48.0% (95% CI: 47.3-48.8) of the population.

### Net ownership at household level

Overall, 59.3% (95% CI: 57.5-61.1) of households had at least one mosquito net, 53.2% (95% CI: 51.4-55.0) had at least one ITN and 50.6% (95% CI: 48.7-52.4) had at least one LLIN. On average, 24.6% (95% CI: 22.3-25.5) of households had at least one mosquito net for every two people (Table 1). The ownership of nets was higher in urban areas than in the rural areas.

**Table 1:** Net ownership by households with at least one mosquito bed net

	**Number of households**	**Having bed net**	**Having ITN**	**Having LLIN**	**Having one net for two people**
**Residency**	N	F (95% CI)	F (95% CI)	F (95% CI)	F (95% CI)
Rural	2,316	57.1 (55.1–59.1)	50.6 (48.7–52.6)	47.9 (45.9–49.9)	23.4 (21.7–25.2)
Urban	481	69.9 (65.6–73.8)	65.7 (61.4–69.8)	63.2 (58.8–67.4)	30.6 (26.8–35.0)
**Region**					
Upper Nile	918	53.7 (50.5–56.9)	42.5 (39.3–45.7)	40.6 (37.5–43.8)	20.6 (18.1–23.3)
Bahr el Ghazal	882	69 (65.9–72.0)	64.6 (61.4–67.7)	59.4 (56.1–62.6)	30.1 (27.2–33.3)
Equatoria	997	55.9 (52.8–58.9)	53 (49.9–56.0)	51.8 (48.8–55.0)	23.5 (20.9–26.2)
**Total**	**2,797**	**59.3 (57.5–61.1)**	**53.2 (51.4–55.0)**	**50.6 (48.7–52.4)**	**24.6 (22.3–25.5)**

N = number, F = Frequency.

### Access and utilization of nets

Overall the proportion of the population with access to an ITN in their household was 49.7% (95% CI: 48.2-51.2). It was higher in urban areas (58.5% (95% CI: 55.0-61.9)) than in rural area (44.8% (95% CI: 43.1-46.5)) and varied by region (Table 2). Generally, only 25.3% (95% CI: 23.9-26.7) of children under five (U5) compared to 35.9% (95% CI: 31.9-40.2) of pregnant women slept under an ITN the night preceding the survey (OR: 1.66 (95% CI: 1.36-2.01); *P* =0.175). Mosquito net usage varied by residency and region (Table 2). Urban children were more likely to sleep under a net (33.2% (95% CI: 29.8-36.8)) than rural children (23.5% (95% CI: 22.0-25.1)); *P* for variation = 0.198. Accordingly, pregnant women in urban areas were less likely than those in rural areas (56.0% (95% CI: 45.8-65.8) *vs* 31.6% (95% CI: 27.3-36.2); *P* = 0.009) to sleep under a mosquito net. Equally, results by region showed differences, with proportions of children U5 and pregnant women who used a mosquito net the night before survey varying from 24.3 and 27.5% in Upper Nile Region to 26.2 and 40.1% in Equatoria Region, respectively (Table 2).

**Table 2:** Access to and use of ITN

	**Population (de-facto)**	**People with access to ITN within Household**	**People who slept under an ITN the previous night**	
			**Children under 5 yrs pregnant women**	
**Residency**	N	% (95% CI)	% (95% CI)	% (95% CI)
Rural	3,445	44.8 (43.1–46.5)	23.5 (22.0–25.1)	31.6 (27.3–36.2)
Urban	781	58.5 (55.0–61.9)	33.2 (29.8–36.8)	56.0 (45.8–65.8)
**Region**				
Upper Nile	1535	35.4 (33.1–37.8)	24.3 (22.2–26.7)	27.5 (21.0–35.2)
Bahr el Ghazal	1186	53.0 (50.2–55.8)	25.7 (23.1–28.6)	38.9 (32.5–45.6)
Equatoria	1505	51.8 (49.3–54.3)	26.2 (24.0–28.7)	40.1 (32.7–48.1)
**Total**	**4,226**	**49.7 (48.2–51.2)**	**25.3 (23.9–26.7)**	**35.9 (31.9–40.2)**

N = number.

### Indoor residual spraying

Only 2% of 2,797 surveyed households had been sprayed in the previous 12 months. Among households sprayed in the previous 12 months, 53.5% (95% CI: 51.6-55.3) also had at least one ITN (Table 3). Heterogeneity was observed in the number of sprayed households by region (Bahr el Ghazal 65% (95% CI: 61.8-68.0) *vs* Upper Nile 43% (95% CI: 39.9-46.3); *P* = 0.034) and also with wealth index (wealthier and urban 69% (95% CI: 65.7-73.8) *vs* poor and rural 45.8% (95% CI: 42.6-49.0); *P* = 0.030).

**Table 3:** Households covered by IRS and those having an ITN

	**Households covered by IRS**		**Household also having ITN**
**Residency**	n (%)	95% CI	% (95% CI)
Rural	2,316 (1.5)	1.09–2.09	50.9 (48.9–52.9)
Urban	481 (4.8)	3.21–7.07	65.9 (60.5–69.0)
**Region**			
Upper Nile	918 (1.0)	0.52–1.85	43.0 (39.9–46.3)
Bahr el Ghazal	882 (4.2)	3.06–5.73	65.0 (61.8–68.0)
Equatoria	997 (1.2)	0.69–2.09	53.0 (49.9–56.0)
**Total**	**2,797 (2.1)**	**1.64–2.71**	**53.5 (51.6–55.3)**

### Level of malaria knowledge

Of the 3,011 women interviewed, 58% (95% CI: 56.2-59.8) reported that malaria is transmitted by mosquitoes, 26.5% (95% CI: 25.0-28.1) said malaria is caused by working in the sun while 12.5% (95% CI: 11.4-13.7) reported that it is caused by drinking dirty water. Additionally, respondents cited different reasons for avoiding getting malaria: sleeping under an ITN, 34.2% (95% CI: 32.5-35.9), taking preventative medicine, 12% (95% CI: 10.9-13.2), spraying the house with insecticide (5%) and destroying mosquito breeding sites (3%). Notably, 41.3% (95% CI: 39.6-43.1) knew the correct treatment for malaria (Table 4).

**Table 4:** Respondents’ knowledge about transmission, prevention and treatment

**Respondents’ awareness**	**Residency**		**Region**			**Total**
	**Rural**	**Urban**	**Upper Nile**	**Bahr el Ghazal**	**Equatoria**	
Malaria transmitted by mosquito bite	53.7%	73.5%	53.3%	60.4%	60.2%	58.0%
	(95% CI: 51.9–55.5)	(95% CI: 71.9–75.0)	(95% CI: 51.5–55.1)	(95% CI: 58.7–62.1)	(95% CI: 58.5–62.0)	(95% CI: 56.2–59.8)
Sleeping under a mosquito nets to prevent malaria	29.8%	50.5%	30.0%	28.0%	43.1%	34.2%
	(95% CI: 28.2–31.5)	(95% CI: 48.7–52.3)	(95% CI: 28.4–31.7)	(95% CI: 26.4–29.6)	(95% CI: 41.4–44.9)	(95% CI: 32.5–35.9)
Taking preventive medicine to avoid malaria	13.3%	7.4%	7.7%	20.9%	8.6%	12%
	(95% CI: 12.1–14.5)	(95% CI: 6.5–8.4)	(95% CI: 6.8–8.7)	(95% CI: 19.5–22.4)	(95% CI: 7.7–9.7)	(95% CI: 10.9–13.2)
Taking the right anti-malaria as treatment	37.8%	54.2%	43.2%	34.1%	44.7%	41.3%
	(95% CI: 36.1–39.5)	(95% CI: 52.4–56.0)	(95% CI: 41.5–45.0)	(95% CI: 32.8 –36.2)	(95% CI: 42.9–46.5)	(95% CI: 39.6–43.1)

N = number, F = Frequency.

### Malaria and anaemia prevalence

**Figure 2:**
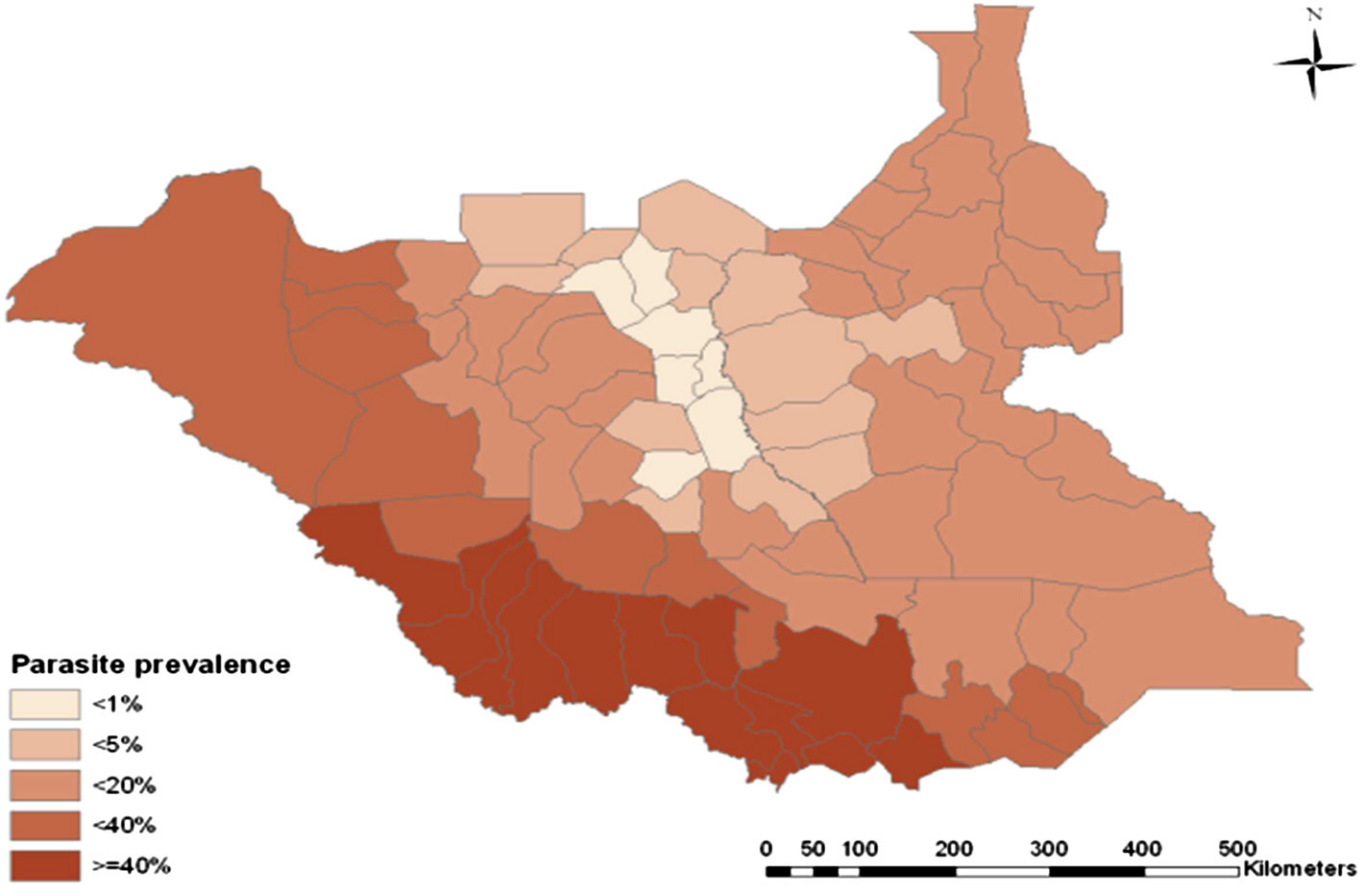
**Map of epidemiological stratification of *Plasmodium falciparum *** **malaria among under fives (Source: MIS 2009).**

Prevalence of infection was 24.5% (95% CI: 23.0-26.1) in children U5 and 9.9% (95% CI: 7.4-13.1) in pregnant women. Overall parasite prevalence ranged from less than 1% to more than 40% with great variability across the states (Figure 2). It was higher in rural areas than in urban areas in both children (25.9% (95% CI: 22.3-29.8) *vs* 18.3% (95% CI: 16.8-19.9); *P* = 0.253) and pregnant women (10.6% (95% CI: 7.8-14.2) *vs* 6.6% (95% CI: 2.8-14.5); *P* = 0.335), respectively. Parasite prevalence was highest in Equatoria region with 47.7% (95% CI: 44.7-50.7) for children and 15.2% (95% CI: 10.2-22.2) for pregnant women. Of the surveyed individuals, 463 tested positive by microscopy with *P. falciparum*, constituting 94.4% of all morbidity, 5% was caused by *Plasmodium vivax,* 0.7% was due to *Plasmodium malariae*, and mixed infections occurred in 6.3% of cases [8]. Almost two thirds (64%) of children U5 were anaemic, 23% mildly anaemic, 30% moderately anaemic, and 11% severely anaemic. Similarly, 46% of pregnant women were anaemic, 23% mildly anaemic, 19% moderately anaemic, and 4% severely anaemic.

### Community access to anti-malaria treatment

Only 35% (95% CI: 33.4-36.7) of children U5 had a fever in the two weeks preceding the survey. The prevalence of fever in children was lowest in Bahr el Ghazal region at 19.1% (95% CI: 13.4-18.0) and highest in Equatoria Region with 52.3% (95% CI: 49.3-55.3). Advice and/or treatment from a health facility or health care provider was sought for 52.0% (95% CI: 48.9-55.1) of the children. Thirty-six per cent took an anti-malaria drug, with only 25% adherence to the national malaria treatment policy. Only 15.0% (12.9-17.3) of children under 5 years who had fever in the two weeks preceding the survey took an AS + AQ, while 8.2% (6.7-10.1) took quinine for the management of the fever. 56.3% (52.2-60.3) of the children were taken to government facilities (hospital, health centre or health unit) for treatment while only 11.4% (9.1-14.3) were taken to a private hospital or clinic (Table 5).

**Table 5:** Fever management and access to anti-malaria treatment by children U5

	**Type of malaria-drugs**			**Source of treatment**		
	**Number of children**	**AS + AQ**	**Quinine**	**Number of children**	**Public sector**	**Private sector**
Residency	N	F(95% CI)	F(95% CI)	N	F(95% CI)	F(95% CI)
Rural	817	15.0(12.8–17.71)	6.7(5.2–8.7)	443	56.2(51.6–60.8)	12.6(9.9–16.1)
Urban	171	15.1(10.6–21.4)	15.6(11.1–22.0)	136	80.4(72.7–86.0)	7.4(4.0–13.0)
Region						
Upper Nile	323	15.4(11.9–19.8)	0.3(0.05–1.73)	137	48.8(71.3–84.4)	2.9(1.1–7.3)
Bahr el Ghazal	145	4.0(1.9–8.7)	0.7(0.12–3.8)	65	41.5(30.4–453.7)	9.2(4.3–18.7)
Equatoria	520	17.8(14.8–21.4)	15.2(12.4–18.5)	377	50.6(45.6–55.7)	14.9(11.6–18.8)
Total	988	15.0(12.9–17.3)	8.2(6.7–10.1)	579	56.3(52.2–60.3)	11.4(9.1–14.3)

N = number, F = Frequency.

## Discussion

Malaria-endemic countries across sub-Saharan Africa are scaling up control efforts in response to the huge disease burden on the continent [5]. Malaria remains a major public health problem in South Sudan and the findings reported here should be interpreted within the context of high malaria transmission areas. LLINs provide personal protection, and, in settings with sustained high levels of coverage and anthropophilic vectors, they can reduce transmission and protect an entire community [14,15]. Most countries have made appreciable progress in scaling up distribution and increasing utilization [5]. This present study demonstrates that both possession of at least one net and net use the previous night before the survey were quite low in South Sudan (Tables 1 and 2). The proportion of the population that could potentially be covered by existing ITNs, assuming that each ITN in a household can be used by two people within that household was higher 49.7% (48.2-51.2) than the utilization rates in both children U5 at 25.3% (23.9-26.7) and pregnant women at 35.9% (31.9-40.2) (Table 2). However, there was no significant difference in use of nets between pregnant women and children U5.

IRS has been advocated by the RBM partnership as a key vector control tool along with LLINs [16,17]. Many malaria-endemic countries have adopted and implemented the intervention with concomitant marked reduction in the disease burden [18,19]. While expansive programmatic deployment of IRS in South Sudan ceased in the 1980s, efforts are being expended towards its implementation in emergency or epidemic situations [1]. Data from this study indicate that only 2% of the surveyed households had been sprayed in the previous 12 months in South Sudan, and exhibit great heterogeneity by regional and residence (Table 3). The observed high level of IRS in urban areas is in line with the amenability of the intervention in urban localities largely due to the logistical ease of deployment compared to the rural localities. On the other front, this could be as a result of ability to pay for the services by the householders, a gesture more likely to be extended by the affluent urban dwellers.

The overall knowledge levels regarding causes of malaria, how to avoid getting malaria and treatment of malaria remain very low among women. This could explain the corresponding low use of malaria control tools observed in the country. The overall malaria parasite prevalence remains very high in South Sudan and requires consistent and expansively scaled-up coverage of effective control tools. As observed in other malaria-endemic settings, the disease exacts its greater toll in the rural areas than in urban areas in both children and pregnant women, with anaemia remaining a public health problem in these vulnerable groups across the country. The study also demonstrated very low levels of access to treatment (Table 5).

The survey was characterized by management and organizational challenges due to limited capacity to conduct the national studies. Of all the women identified in the household questionnaires as eligible, only about 80% were interviewed. The blood slides used in the survey were not bar-coded thus labeling was prone to human error resulting in mismatching numbers. The eight-month period between data collection and data processing, due to logistical challenges, was too long and might have contributed to missing information and compromise of the quality of data. Although RDTs yielded more positive cases of infection than microscopy, they have the potential to give false positives due to the detection of HRP-2 antigens following a previously treated infection for up to several weeks [11]. Due to limitations in conclusive data, this study could not take into consideration complete analysis of The two additional RBM indicators of “proportion of households with at least one ITN for every two people” and “proportion of population with access to an ITN within the household” as recommended by MERG. Including the inherent gaps: i) households with no ITN, ii) households with any but not enough ITN, iii) population with access to ITN not using it [20].

This nationwide malaria-specific survey has demonstrated progress in the coverage of interventions relative to the household surveys conducted in previous studies in South Sudan [3,21]. This information is useful to the stakeholders for implementing decisions on malaria programming in South Sudan. The generated malaria parasite prevalence data have been used to develop the first ever malaria epidemiological maps for the country (Figure 2). This has provided the empirical basis for policy decision-making by the NMCP and guiding deployment of malaria control interventions across the nation.

High levels of parasite prevalence have consistently been reported by population-based surveys conducted in different sub-Saharan African countries [22-24]. The present findings corroborate the results of these earlier studies. Ownership of LLINs has been reported to be relatively high than their utilization that still remains relatively low [23,25]. Like many other countries, the implementation of IRS in South Sudan is also minimal (Table 3). The low number of households that had been covered by intradomiciliary spraying the year preceding the survey is consistent with findings from Djibouti [25], Angola [22] and Ethiopia [23] but considerably lower than those reported from Zambia [20]. Though Zambia has a more comprehensive spraying programme than South Sudan and the other countries cited, this could be ascribed to unsuitability of housing structures at the time of the survey in South Sudan. The situation is improving steadily and the intervention could be implemented if requisite resources are made available. Other than for Zambia, the low levels of knowledge on malaria control and prevention mirror those of other surveys in sub-Saharan African countries [23-25]. Unlike the experiences in other surveys where net ownership and use are higher in rural compared to urban areas [24], in this study the converse was true with possession and use of nets being higher in urban areas than in rural areas though there are countries or sub national areas where the opposite is true. This could be ascribed to increasing levels of urbanization and the inherent urban drift of the population coupled with an increased access to information, education and behavioural change communication (IEC/BCC) messages. The low levels of treatment are also consistent with what has been reported before by other countries in the region [23-25].

The findings in this study substantiate the need for consistency in conducting population-based health surveys in order to generate empirical evidence for decision-making. Recognizing that low coverage of LLINs cannot afford the desired impact, particularly when utilization remains minimal, there is need to step up distribution to meet the minimum 80% coverage target to afford community effect. Universal coverage of all households with adequate numbers of mosquito nets is critical to increase access and utilization, particularly by children and pregnant women. This would require investment in extensive IEC/BCC campaigns to raise the awareness of communities on the importance of using ITNs for malaria prevention. The national coverage for IRS in South Sudan falls far short of the 80% household coverage target required to offer vector control benefits [17]. However, the 80% target has been attained in the very limited areas that were targeted for IRS in South Sudan with strong NGO support and substantiates the need for large-scale implementation. With very low early treatment-seeking behaviour, that falls well below national and international targets, training of more health workers, including those at community level, will be critical in improving the access to treatment and vector control interventions. South Sudan has shown that with strong leadership and strengthened collaboration, it is possible for the malaria control programme to rapidly scale up its full intervention package, even in socially, economically challenged conflict and post-conflict settings.

## Conclusions

The observed high malaria prevalence could be due to low levels of coverage and utilization of interventions as well as low knowledge levels. Access, coverage and utilization of malaria control tools should be increased through scaling up interventions and improving behavioural change communication. Periodic stand-alone MISs will go a long way in informing policy decision making. Investment should be made in strengthening the human resource capacity within the NMCP and the other departments of the MoH in conducting and managing surveys.

## Abbreviations

ACT: Artemisinin-based combination therapy; BCC: Behaviour change communication; DFID: Department for International Development; EA: Enumeration area; IEC: Information, education and communication; IPT: Intermittent preventive treatment; IRS: Indoor residual spraying; ITNs: Insecticide-treated nets; LLINs: Long-lasting insecticidal nets; MERG: Monitoring and evaluation reference group; MIS: Malaria indicator survey; MoH: Ministry of Health; NMCP: National malaria control programme; RBM: Roll back malaria; RDTs: Rapid diagnostic tests; SSCCSE: Southern Sudan centre for census statistics and evaluation; ToT: Training of trainers; UNDP: United Nations Development Programme; UNICEF: United Nations international children emergence fund; USAID: United States Agency for International Development; WHO: World Health Organization.

## Competing interests

The authors declare that they have no competing interests.

## Authors’ contributions

The designing, planning and implementation including data management, analysis and report writing for this first ever Malaria Indicator Survey in South Sudan was a collaborative effort by multiple individuals from local and international malaria stakeholders. MBE led and coordinated the survey and reviewed the manuscript. AJ, CDR, BS, ZA, OT, EB, RLL, and LR collaborated. EC conceived the idea and wrote the paper. All authors read and approved the final manuscript.
